# Genetic and Functional Dissection of *HTRA1* and *LOC387715* in Age-Related Macular Degeneration

**DOI:** 10.1371/journal.pgen.1000836

**Published:** 2010-02-05

**Authors:** Zhenglin Yang, Zongzhong Tong, Yuhong Chen, Jiexi Zeng, Fang Lu, Xufang Sun, Chao Zhao, Kevin Wang, Lisa Davey, Haoyu Chen, Nyall London, Daisuke Muramatsu, Francesca Salasar, Ruben Carmona, Daniel Kasuga, Xiaolei Wang, Matthew Bedell, Manjuxia Dixie, Peiquan Zhao, Ruifu Yang, Daniel Gibbs, Xiaoqi Liu, Yan Li, Cai Li, Yuanfeng Li, Betsy Campochiaro, Ryan Constantine, Donald J. Zack, Peter Campochiaro, Yinbin Fu, Dean Y. Li, Nicholas Katsanis, Kang Zhang

**Affiliations:** 1 Center for Human Molecular Biology and Genetics, Sichuan Academy of Medical Sciences and Sichuan Provincial People's Hospital, Chengdu, Sichuan, China; 2 Institute for Genomic Medicine and Shiley Eye Center, University of California San Diego, San Diego, California, United States of America; 3 Department of Ophthalmology and Visual Sciences, Moran Eye Center, University of Utah School of Medicine, Salt Lake City, Utah, United States of America; 4 Institute of Genetic Medicine, Johns Hopkins University, Baltimore, Maryland, United States of America; 5 Program in Human Molecular Biology and Genetics, Eccles Institute of Human Genetics, University of Utah School of Medicine, Salt Lake City, Utah, United States of America; 6 Wilmer Eye Institute, Johns Hopkins University, Baltimore, Maryland, United States of America; 7 Departmet of Ophthalmology, Xin Hua Hospital, Shanghai Jiao Tong University, Shanghai, China; 8 Laboratory of Analytical Microbiology, Institute of Microbiology and Epidemiology, Beijing, China; 9 Department of Molecular Biology and Genetics, Johns Hopkins University, Baltimore, Maryland, United States of America; 10 Veteran's Administration, San Diego, California, United States of America; 11 Department of Ophthalmology and Ophthalmic Laboratories, West China Hospital, Sichuan, China; Stanford University School of Medicine, United States of America

## Abstract

A common haplotype on 10q26 influences the risk of age-related macular degeneration (AMD) and encompasses two genes, *LOC387715* and *HTRA1*. Recent data have suggested that loss of *LOC387715*, mediated by an insertion/deletion (in/del) that destabilizes its message, is causally related with the disorder. Here we show that loss of *LOC387715* is insufficient to explain AMD susceptibility, since a nonsense mutation (R38X) in this gene that leads to loss of its message resides in a protective haplotype. At the same time, the common disease haplotype tagged by the in/del and rs11200638 has an effect on the transcriptional upregulation of the adjacent gene, *HTRA1*. These data implicate increased *HTRA1* expression in the pathogenesis of AMD and highlight the importance of exploring multiple functional consequences of alleles in haplotypes that confer susceptibility to complex traits.

## Author Summary

Age-related macular degeneration (AMD) is the leading blindness cause in western countries. Several genes encoding components of the complement pathway—including *CFH*, *C2/BF*, and *C3*—have been confirmed to be associated with AMD, as well as a region on 10q26 that encompasses two genes. Recent data have suggested that loss of *LOC387715* on 10q26, mediated by an insertion/deletion (in/del) at its 3'UTR that destabilizes its message, is causally related with the disorder. We found that a common disease haplotype including the in/del and rs11200638 also has an effect on the transcriptional upregulation of the adjacent gene, *HTRA1*. We propose a binary model where downregulation of *LOC387715* and concomitant upregulation of *HTRA1* best explain the risk associated with the 10q26 AMD region.

## Introduction

Genome-wide association studies (GWAS) have catalyzed significant progress towards elucidating the molecular basis of complex traits [1]. However, a substantial gap remains between association of a trait with a genomic segment and the identification of the causative allele(s). A locus for AMD, ARMS2 on 10q26, illustrates this challenge.

ARMS2 is one of several regions in the genome shown by GWAS to confer susceptibility to the disorder. However, in contrast to the other confirmed AMD susceptibility loci *CFH* (NM_000186) [2]–[8], *C2/BF* (NM_000063/NM_001710) and *C3* (NM_000064) [9]–[11], which encompass a single gene; a region on 10q26 that contains three genes has been associated consistently with AMD susceptibility [12]–[15]. Recent data have refined this association to a haplotype that encompasses two genes, *LOC387715* (NM_001099667), a primate-specific transcript with a proposed mitochondrial function [16] and *HTRA1* (NM_002775), a multi-functional serine protease [17]–[18].

Initial reports describing the association between SNP rs11200638 in the 5′ end of *HTRA1* and AMD focused on the possibility that the disease-associated allele of this SNP increased expression of HTRA1 [17]–[18]. However, the inability to verify this finding in heterologous expression systems led to the investigation of other alleles in the risk haplotype, and emphasis was placed on *LOC387715* [16]. Consistent with this possibility, recent sequencing of the AMD-associated haplotype identified multiple SNPs, including an in/del, that are associated with an increased risk of AMD, and a decreased *LOC387715* mRNA level with this AMD disease haplotype [19]. To delineate the causal genetic variations contained in the risk haplotype and to understand their functional roles in AMD susceptibility, we undertook genetic and functional investigations at the ARMS2 locus. We show that loss of *LOC387715* is likely necessary but not sufficient to explain AMD susceptibility and that a common disease haplotype including the in/del and rs11200638 also has an effect on the transcriptional upregulation of the adjacent gene, *HTRA1*. These data, which implicate increased HTRA1 expression in the pathogenesis of AMD, suggest that the AMD risk conferred by this region is potentially driven by multiple variants, and highlight the importance of exploring multiple functional consequences of alleles in haplotypes that confer susceptibility to complex traits.

## Results

Several studies have shown previously strong association of multiple single nucleotide polymorphisms (SNPs) in chromosome 10q26 encompassing *PLEKHA1*, *LOC387715*, and *HTRA1*with advanced AMD [12]–[24]. From our previous study [20], we identified a common disease haplotype TAT tagged by rs10490924, rs11200638 and rs2293870 that is significantly associated with a risk of AMD (*P* = 2.70×10^−9^), as well as a haplotype GGG that is modestly, yet significantly, associated with protection from AMD (P = 0.003). Next, to define the extent of the haplotype structures and capture all variants in the region, we undertook a re-sequencing effort of a region spanning ∼100 kb, starting in the 3′-UTR of *PLEKA* and ending ∼20kb downstream of the 3′ UTR of *HTRA1* in six individuals with a homozygous risk haplotype and six individuals with a homozygous protective haplotype, followed by assessment of association of each discovered variant with AMD in 200 AMD cases and 200 normal controls (Table 1, Table S1, Table S2).

**Table 1:** Association study for SNPs in 10q26 region in 200 cases and 200 controls in the Utah cohort.

SNP	Chromosome Position	Risk Allele	Case MAF	Control MAF	Trend p-Value
SNP-2 (New SNP)	124198539-124198544	del 6bp	0.40	0.27	5.96×10^−5^
rs11200630	124199674	C	0.39	0.25	8.95×10^−6^
ENSSNP6019764	124200359	A	0.39	0.21	5.86×10^−6^
rs10490924	124204438	T	0.41	0.23	4.90×10^−7^
SNP-3 (New SNP)	124204546-124204547	ins GT	0.49	0.37	3.75×10^−5^
rs36212731	124204966	T	0.30	0.20	2.47×10^−3^
rs36212732	124205188	G	0.42	0.26	6.64×10^−6^
rs36212733	124205201	C	0.41	0.25	4.85×10^−7^
rs3750848	124205305	G	0.41	0.23	1.23×10^−6^
rs3750847	124205411	T	0.35	0.24	6.43×10^−4^
rs3750846	124205555	C	0.45	0.24	5.87×10^−3^
SNP-4 (New SNP)	124206332-124206333	ins AT	0.69	0.61	0.02
in/del/Wt	124206811-124207253	ins 54bp, del 443bp	0.40	0.22	2.66×10^−7^
SNP-5 (New SNP)	124207752-124207753	ins G	0.48	0.33	5.81×10^−5^
rs3037985	124207893	del 7bp	0.68	0.59	0.03
rs3793917	124209265	G	0.40	0.22	3.22×10^−6^
rs3763764	124210051	C	0.41	0.21	2.38×10^−7^
rs3763764	124210051	G	0.46	0.30	4.20×10^−6^
rs11200638	124210534	A	0.41	0.22	2.19×10^−7^
rs1049331	124211260	T	0.43	0.29	2.68×10^−3^
rs2293870	124211266	T, C	0.53	0.32	5.56×10^−7^
rs58077526 or rs61871752	124220014	C	0.38	0.21	2.17×10^−6^

**Figure 1:**
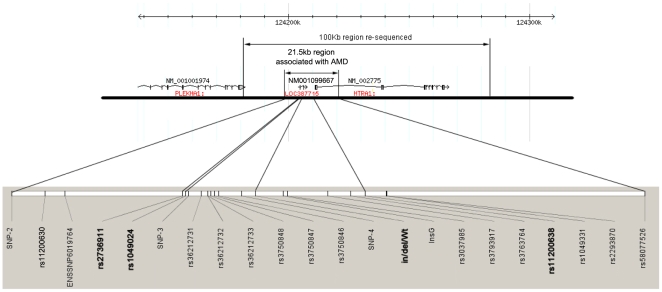
Genes and the main SNPs in the AMD 10q26 region. The schematic diagram showing a 100 kb region subject to re-sequencing, the AMD-associated 21.5 kb region containing 22 SNPs in the same LD block with a disease haplotype. SNPs in bold were chosen for further studies.

In agreement with recent work [19], we discovered that both risk and protective haplotypes span 21.5kb in the *ARMS2* region, starting from upstream of *LOC387715* to rs58077526, in intron 1 of *HTRA1* (Figure 1, Table 1, Table S1). We confirmed recent data [19], according to which 22 SNPs tagged a risk and a protective haplotype including previously reported SNPs rs10490924, rs3750848, an in/del, rs3793917, and rs11200638 (Figure 1 and Table 1). Moreover, the reported 54 base pair insertion and 443 base pair deletion (in/del) at the 3′ end of *LOC387715* resides exclusively on the disease risk haplotype that is strongly associated with a risk of AMD (*P* = 1.90×10^−26^) in a Utah case-control cohort (705 cases, 650 controls) (Table 2 and Table 3), as well as an independent replication cohort of northern European ancestry (442 cases, 434 controls ) (Table 2 and Table 3) and a third replication cohort of Han Chinese in China (138 cases, 591 controls ) (Table 2 and Table 3) [19].

**Table 2:** Association of rs2736911, rs10490924, in/del/Wt, and rs11200638 in the three independent cohorts.

SNP	Case-control cohort	Utah cohort	Hopkins cohort	Han Chinese cohort
	Case # vs Control #	705 vs 650	442 vs 434	138 vs 591
**rs2736911**	Allelic p-value	6.46×10^−5^	1.80×10^−3^	0.011
	Heterozygote OR (95% CI)	0.611(0.475–0.788)	0.655(0.471–0.909)	0.411(0.236–0.715)
	Homozygote OR (95% CI)	0.497(0.2233–1.107)	0.254(0.052–1.232)	2.48(0.410–15.031)
**rs10490924**	Allelic p-value	8.61×10^−26^	4.87×10^−34^	1.15×10^−13^
	Heterozygote OR (95% CI)	2.069(1.645–2.603)	3.175(2.333–4.321)	2.664(1.284–5.527)
	Homozygote OR (95% CI)	7.191(4.518–11.444)	10.311(6.474–16.422)	8.986(4.335–18.625)
**in/del/Wt**	Allelic p-value Freq.	1.90×10^−26^	8.35×10^−34^	6.03×10^−13^
	Heterozygote OR (95% CI)	2.305(1.827–2.908)	3.195(2.339–4.364)	2.395(1.189–4.825)
	Homozygote OR (95% CI)	6.879(4.436–10.666)	7.998(5.255–12.173)	7.983(3.965–16.072)
**rs11200638**	Allelic p-value	3.64×10^−26^	2.52×10^−34^	5.10×10^−13^
	Heterozygote OR (95% CI)	2.413(1.922–3.031)	3.315(2.429–4.523)	4.087(1.602–10.425)
	Homozygote OR (95% CI)	6.851(4.396–10.676)	10.246(6.550–16.026)	12.932(5.083–32.904)

**Table 3:** Genotyping results of rs2736911, rs10490924, in/del/Wt, and rs11200638 in the three independent cohorts.

SNP	Case-control cohort	Utah case-control cohort		Hopkins case-control cohort		Han Chinese case-control cohort	
	Groups	Case	Control	Case	Control	Case	Control
	Number	705	650	442	434	138	591
rs2736911	Protective Allele T Freq.	0.12	0.18	0.09	0.14	0.07	0.12
	HWE	1.00	0.45	0.50	0.71	0.11	0.29
rs10490924	Risk Allele T Freq.	0.38	0.20	0.25	0.24	0.74	0.49
	HWE	0.05	0.34	0.66	0.80	0.07	0.99
in/del/Wt	Risk Allele in/del Freq.	0.39	0.20	0.53	0.25	0.73	0.49
	HWE	0.06	0.88	0.12	0.25	0.08	1.00
rs11200638	Risk Allele A Freq.	0.41	0.22	0.53	0.25	0.77	0.52
	HWE	0.38	0.22	0.10	1.00	0.05	0.28

**Figure 2:**
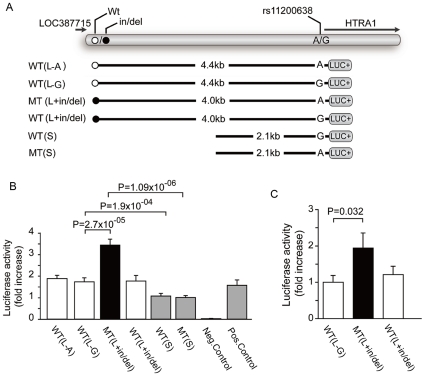
The haplotype block in D' of rs2736911, rs10490924, in/del/Wt, and rs11200638 in the Utah case-control cohort. The risk haplotype C-T-in/del-A is strongly associated with advanced AMD (*P* = 4.05×10^−28^); the non-risk haplotype T-G-Wt-G is significantly associated with protection of advanced AMD (*P* = 4.00×10^−4^). In/del and Wt refers to presence or absence of 54 base pair insertion and 443 base pair deletion, while the letters G, T, A refer to nucleotides for the respective SNPs at rs2736911, rs10490924, and rs11200638.

In contrast to the loss of function role of in/del in *LOC387715*, the T allele of SNP rs2736911, a non-synonymous coding SNP leading to a predicted premature stop (R38X) in *LOC387715* is associated with a protective haplotype T-G-Wt-G (*P* = 4.00×10^−4^, Figure 2 and Table 2 and Table 4) defined by rs2736911, rs10490924, in/del/Wt and rs11200638. We replicated these findings twice and found that this haplotype is clearly not associated with AMD risk; rather it is (conservatively) neutral and possibly protective with regard to the disorder (*P* = 0.001 in the second replication cohort; *P* = 0.007 in third Han Chinese cohort, Table 4).

**Table 4:** Haplotype structures generated by the Hapview program using rs2736911, rs10490924, in/del/Wt, and rs11200638.

Case-control cohort	Haplotype	Case Freq.	Control Freq.	Chi Square	P Value
**Utah case-control cohort**	C-G-Wt-G	0.450	0.590	50.455	1.22×10^−12^
	C-T-in/del-A	0.340	0.160	120.883	4.05×10^−28^
	T-G-Wt-G	0.110	0.150	12.723	4.00×10^−4^
	C-G-Wt-A	0.028	0.024	0.559	0.455
	C-T-in/del-G	0.014	0.018	0.602	0.438
	T-G-Wt-A	0.009	0.019	4.621	0.032
	C-T-Wt-A	0.003	0.009	3.576	0.059
**Hopkins case-control cohort**	C-G-Wt-G	0.370	0.580	76.762	1.93×10^−18^
	C-T-in/del-A	0.500	0.210	167.657	2.40×10^−38^
	T-G-Wt-G	0.076	0.120	10.773	0.001
	C-G-Wt-A	0.017	0.022	0.708	0.400
	C-T-Wt-G	0.010	0.021	3.752	0.053
	C-G-in/del-G	0.006	0.018	5.261	0.022
	T-T-in/del-A	0.014	0.007	2.085	0.149
**Han Chinese case-control cohort**	C-G-Wt-G	0.170	0.330	27.937	1.25×10^−7^
	C-T-in/del-A	0.660	0.450	62.000	3.43×10^−15^
	T-G-Wt-G	0.037	0.084	7.376	0.007
	C-G-in/del-A	0.040	0.028	0.992	0.319
	C-T-Wt-G	0.023	0.032	0.620	0.431
	C-G-Wt-A	0.016	0.031	1.874	0.171
	C-T-Wt-A	0.015	0.028	1.502	0.220
	T-T-in/del-A	0.017	0.018	0.006	0.940
	C-T-in/del-G	0.009	0.014	0.482	0.488
	C-G-in/del-G	0.001	0.014	3.488	0.062

**Figure 3:**
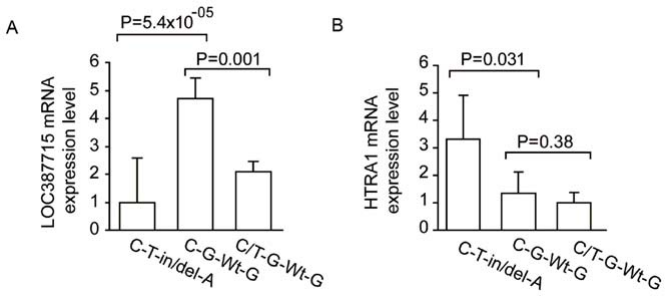
Endogenous expression studies comparing effects of genotype on *HTRA1* and *LOC387715*. mRNA Expression levels of *LOC387715* and *HTRA1* in human placenta tissues according to haplotypes defined by SNPs rs2736911, rs10490924, in/del/Wt, and rs11200638. Human placenta tissues from seven individuals with a homozygous disease haplotype (C-T-in/del-A), five individuals with a homozygous protective haplotype (C-G-Wt-G), and five individuals with heterozygous C/T alleles at rs2736911 and homozygous protective haplotype at rs10490924, in/del/Wt, and rs11200638 (C/T-G-Wt-G) were harvested and analyzed. (A) In comparison to the haplotype C-G-Wt-G, mRNA levels of *LOC387715* with a homozygous disease haplotype C-T-in/del-A and a haplotype C/T-G-Wt-G were 4.7-fold and 2.3 fold lower, respectively. The mean ± SD is given for each genotype. (B) mRNA levels of *HTRA1* with C-T-in/del-A was 2.7-fold higher compared to that of C-G-Wt-G, and there was comparable expression levels between C-G-Wt-G and C/T-G-Wt-G. The mean ± SD is given for each genotype.

These findings present a paradox. Recent studies have shown the in/del to cause destabilization of *LOC387715*, suggesting that loss of function at that locus might confer risk to AMD. However, the introduction of the R38X mutation, which is also predicted to give rise to loss of *LOC387715* message due to nonsense-mediated mRNA decay (NMD), is mildly protective. We considered two plausible alternatives; either that effect of R38X and the in/del have different effects on the transcript, or that loss of *LOC387715* is insufficient to cause AMD. The first possibility is unlikely. Upon quantification of *LOC387715* mRNA levels in seven placentas homozygous for the major disease haplotype, we found a 4.7-fold reduction in endogenous *LOC387715* expression (Figure 3A), in agreement with recent published data [19]. However, we were also able to examine *LOC387715* mRNA levels in five patients heterozygous for the R38X mutation, where we observed a 50% reduction (Figure 3A), suggesting that each of the R38X and in/del have the same mode of action.

Given these data, we considered the potential role of *HTRA1*, the only other locus encompassed by the major risk haplotype, in the pathogenesis of AMD. We have shown previously a three-fold increase expression of *HTRA1* in the retinal pigmented epithelium (RPE) of patients with the risk haplotype [17], while others have also observed increased *HTRA1* expression in AMD eyes in humans [25]. In addition, a functional SNP in the promoter region of *HTRA1* is associated with increased *HTRA1* expression in non-human primates with AMD-like phenotype [26]–[27]. We therefore expanded our analyses of mRNA levels in human placentas to determine the effect of a haplotype on endogenous expression. We found that, in addition to the observed decrease of *LOC387715* message, the disease haplotype is also associated with a 2.7-fold increase in *HTRA1* expression (Figure 3B). These data confirmed that the in/del or another component of the common risk haplotype affects *LOC387715* mRNA stability. At the same time, it likely has an effect on HTRA1 expression. Although caution should be exercised when translating findings from one tissue to another, these data from placenta should serve as an indication for the effects of different haplotypes on the gene expression profiles in the eye.

**Figure 4:**
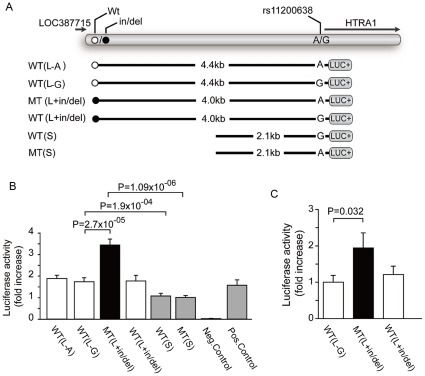
Heterologous Luciferase assays *in vitro* and *in vivo*. Effects of the in/del variants on *HTRA1* expression in cultured human RPE cells and mouse RPE *in vivo*. (A) Schematic diagram of constructs for luciferase reporter assays. The L and the S denote long and short promoter constructs, A/G represents the allele at rs11200638, while in/del (black circle) refers to long promoter construct with an in/del. Different *HTRA1* promoter sequences corresponding to risk and wild-type alleles of in/del and rs11200638 (WT(L-A)), WT(L-G), (MT(L+in/del)), (WT(L+in/del), WT(S) and MT(S)) were placed upstream of a pGL3 reporter. (B) Luciferase activities in cultured human RPE cells transfected with different *HTRA1* promoter reporter constructs. The pGL3-Basic vector without insert (negative) was transfected into human RPE cells as a negative control. Renilla luciferase plasmid pTK-RL was cotransfected with each construct as an internal control for normalization. Normalized luciferase activity was measured in five independent experiments. The mean ± SD is given for each construct. (C) Luciferase activities in mouse RPE *in vivo* corresponding to different HTRA1 promoter reporter constructs. Reporter constructs were injected into the subretinal space and electroporated into mouse RPE cells. Eighteen, nineteen and sixteen eyes were injected with WT(L), MT(L+in/del) and WT(L+in/del) constructs respectively. The firefly luciferase value was divided by the Renilla luciferase value to give the normalized luciferase activity. This was divided by the normalized luciferase activity of empty-PGL3 and pRL-CMV to give the relative luciferase activity ratio. Each bar represents the mean (± SEM).

To test this hypothesis further, we generated expression constructs that model the effect of the risk and protective haplotype on the *HTRA1* promoter using a luciferase reporter assay on cultured human retinal pigment epithelial cells (Figure 4A and 4B). We observed a two-fold increase in luciferase expression in constructs that modeled the disease haplotype encompassing the in/del and the A allele of SNP rs11200638 (MT(L+in/del), Figure 4A and 4B). We detected no increase in luciferase expression from constructs that contained a short disease haplotype including the A risk allele of SNP rs11200638 but did not contain the in/del (MT(S), Figure 4A and 4B). These findings are consistent with Fritsche et al. and Kanda et al [16],[19], who reported an inability to verify that the A allele of SNP rs11200638 by itself alters transcriptional activity in heterologous cell systems. We did not observe increased luciferase expression either when we placed the in/del or A allele of SNP rs11200638 on a construct containing a protective haplotype, suggesting that the in/del or A allele of SNP rs11200638 by itself is insufficient to drive HTRA1 expression (WT(L+in/del), WT(L-A), Figure 4B).

Because of the potentially ambiguous nature of *in vitro* luciferase assays, we repeated these experiments in an *in vivo* system, whereby the constructs containing the disease haplotype including the in/del and the protective haplotype described earlier (Figure 4A) were electroporated into the RPE of wild-type adult mice. Luciferase activity was assayed four days after transfection. Consistent with the *in vitro* data, we observed a significant increase in normalized luciferase activity in a construct bearing the disease haplotype tagged by the in/del and A allele of SNP rs11200638 (*P* = 0.032) (Figure 4C), whereas no increased luciferase activity was observed in a construct with the in/del on a protective haplotype (*P* = 0.180).

## Discussion

The AMD-associated region on 10q26 poses an interesting problem. Multiple lines of evidence support the causality of both *LOC387715* and *HTRA1* in conferring risk to AMD. At the same time, emerging data suggests that mutations in either gene alone might be insufficient to confer such risk. The association signal is best explained by a common disease haplotype including an in/del in the 3′ UTR of *LOC387715* and the A allele of SNP rs11200638. Although there is evidence that AMD patients exhibit loss of *LOC387715* message, the fact that haploinsufficiency in *LOC387715* alone (through the R38X mutation) does not confer risk to AMD, suggests that either that transcript is unrelated to the disorder, or that additional events within the risk haplotype must occur. Although we cannot formally reject the hypothesis that loss of *LOC387715* is irrelevant to the disease, the spatiotemporal expression pattern of this gene and its exclusive emergence with the evolution of the macula in non-human primates, provide partial evidence for its role in AMD pathogenesis [14],[16],[19],[26]. At the same time, the evidence for the involvement of *HTRA1* upregulation is likewise compelling, including i) the upregulation of the transcript in the major risk haplotype, and ii) the observed increased *HTRA1* expression in AMD eyes.

One can outline reasonable hypotheses to explain each aspect of the discrepant data. Alternatively, a single hypothesis of dual causality might explain all the observations. Specifically, we speculate that concomitant downregulation of *LOC387715* and upregulation of *HTRA1* can explain the disease. This theoretical binary model is consistent with the fact that i) the common AMD-associated haplotype affects both transcripts and ii) the protective haplotype containing the R38X *LOC387715* allele is associated with normal *HTRA1* expression (Figure 3B). Moreover, we suggest that if changes in either gene alone were sufficient to confer AMD susceptibility, one might have expected to discover rare alleles that recapitulate the effect of the in/del (such as rare loss of function mutations in *LOC387715* or activating mutations in *HTRA1*), neither of which have been discovered to date by us or by other groups. The primate-specific nature of *LOC387715* renders this binary model intractable in model organisms. Nonetheless, this model may be tested in the monkey using genetic manipulations.

## Materials and Methods

### Patients

This study was approved by the Institutional Review Boards of the University of Utah, University of California San Diego, Johns Hopkins University, and Sichuan Provincial People's Hospital. Subjects gave informed consent prior to participation. Participants underwent a standard examination, which included visual acuity measurements, dilated slit lamp biomicroscopy, and stereoscopic color fundus photography. Grading was carried out with the classification established by AREDS [28]. Diagnosis of advanced AMD was based on the presence of GA or CNV (equivalent to AREDS category 4 or 5). Control subjects were >60 years of age, with no signs of AMD, defined as no drusen or RPE abnormalities in the Utah collection, and controls in the Hopkins cohort and the Chinese cohort were defined as being >60 years old, having fewer than 5 small drusen (<63 um), and no RPE abnormalities. Patient characteristics of the case-control series are listed in Table 5.

**Table 5:** Disease status, gender, and age of subjects in the study.

Case-control cohort		Utah case-control cohort			Hopkins case-control cohort			Han Chinese case-control cohort	
		GA	CNV	Controls	GA	CNV	Controls	CNV	Controls
		232	473	650	141	301	434	138	591
Sex-Number (%)	Female	139 (60)	243 (50)	400 (62)	80 (57)	163 (54)	228 (53)	66 (48)	272 (46)
	Male	93 (40)	230 (50)	250 (38)	61 (43)	138 (46)	206 (47)	72 (52)	319 (54)
Average Age-years		84.09	82.85	75.30	82.51	79.51	78.10	68.10	66.50

### Genotyping

SNPs were genotyped by SNaPshot on an ABI 3100XL analyzer (ABI, Foster City, CA, USA) according to the manufacturer's instructions. PCR and SNaPshot primers are listed in Table S2. All SNPs had a genotyping success rate >98% and accuracy >99% as judged by random re-sequencing of 20% of samples in all three case-control series.

### Re-sequencing of haplotypes

To discover all variations in the AMD susceptibility locus at 10q26, we undertook a re-sequencing of 100kb in the region including genes *PLEKHA1*, *LOC387715* and *HTRA1*. We chose DNA samples from six individuals with a homozygous risk haplotype and six individuals with a homozygous protective haplotype for re-sequenbcing analysis. We designed PCR primer pair for amplification of 1kb amplicon; each amplicon had a 50bp overlap with next amplicons. The PCR amplicons were directly purified using a Qiaquick kit (Qiagen, Valencia, CA, USA) and sequenced using forward and reverse primers using the BigDye Terminator v3.1 Cycle Sequencing Kit (ABI, Foster City, CA, USA) according to the manufacturer's instructions. Then the sequencing results were annotated using the NCBI genomic DNA sequence information (http://www.ncbi.nlm.nih.gov, build 36).

### Statistical analysis

All SNP genotyping results were screened for deviations from Hardy-Weinberg equilibrium with no SNP showing significant deviation (p>0.05). The chi-squared test for allelic trend for an additive model or dominant allele model over alleles was performed with PEPI version 4.0 [29]. Odds ratios and 95% confidence intervals were calculated by conditional logistic regression with SPSS version 13.0. Linkage disequilibrium (LD) structure was examined with Haploview (version 4.0) [30]. Default settings were used, creating 95% confidence bounds on D' to define pair-wise SNPs in strong LD [31]. Haploview was also used for allelic association tests.

### Endogenous expression levels of *HTRA1* and *LOC387715*

Total RNA was extracted from human placentas, and the first strand cDNA was generated by reverse transcript using reverse transcript kit (Invitrogen, Carlsbad, CA, USA). Real time PCR was performed for *HTRA1* mRNA qualification using ABI human *HTRA1* probe real time PCR kit (ABI, Foster city, CA, USA). RT-PCR was performed to qualify *LOC387715* mRNA levels using primers 5′-atggcagctggcttggcc-3′ and 5′-ttgctgcagtgtggatgatag-3′ with ex taq polymerase (TaKaRa Bio USA, Mountain View, CA USA). *GAPDH* was used as the internal control. Human placenta tissues were harvested and genotyped. We measured RNA levels in seven individuals with a homozygous disease haplotype (C-T-in/del-A, Figure 3A and 3B), five individuals with a homozygous protective haplotype (C-G-Wt-G, Figure 3A and 3B), and five individuals with a homozygous non-risk haplotype with respect to in/del/Wt and a heterozygous CT allele at rs2736911 (C/T-G-Wt-G, Figure 3A and 3B). Significance was examined using SPSS's independent sample t-test.

### Heterologous Luciferase assays

A 4423 bp DNA fragment containing the −1 to −4423 position from the *HTRA1* translation start site including either the risk haplotype (MT(L+in/del), Figure 4A) or protective haplotype (WT(L-G), Figure 4A) was PCR amplified from genomic DNA of an individual with a homozygote risk haplotype or an individual with a protective haplotype using the following primers: forward: cgacgcgtcggatgcagccaatcttctcctaac; reverse: agatctcgagcccggcgactctggcggcggcggcggtg). A DNA fragment containing −1 to 2100bp from the HTRA1 translation site including either the risk haplotype (MT(S), Figure 4A) or protective hapotype (WT(S), Figure 4A) was amplified from genomic DNA of an individual with a homozygous risk haplotype or protective haplotype using the primers, cggggtaccaactcctgggctcaaaggat and ccgctcgagtccgcgcctggccggggtccctcag. Constructs were subcloned into the Mlu I-Xho I site of the pGL3-basic vector (Promega, Madison, WI, USA). All constructs were verified by restriction enzyme digestion and complete bidirectional DNA sequencing. The wild type haplotype constructs carrying in/del (WT(L+in/del), Figure 4A)was generated by subcloning of a fragment containing in/del cutting from MT(L+in/del) using Mlu I-Nhe I. The wild type haplotype constructs carrying A allele at rs11200638 (WT (L-A), Figure 4A) was generated using site-directed mutagenesis kit (Agilent Technologies, La Jolla, CA). A positive control plasmid (pGL3-control Vector) containing an SV40 enhancer and promoter driving luciferase reporter was obtained from Promega (Madison, WI, USA).

Human RPE cells were split into 24-well plates and cotransfected 24 hours later with 1ng of the transfection control Renilla luciferase plasmid pTK-RL (Promega, Madison, WI) and 200ng of one of the following plasmids: pGL3-basic, pGL3-control, WT(L-A), WT(L-G), WT(L+in/del), MT(L+in/del), WT(S) and MT(S). Transfections (n = 6, three preps for each construct and 2 transfections for each preps) were done using the Fugene-6 protocol according to manufacturer's specifications (Roche Applied Science, Mannheim, Germany). Forty-eight hours after transfection, cells were washed with PBS twice and luciferase activities measured with the Dual-Luciferase Assay Kit (Promega, Madison, WI). Fold induction was derived relative to normalized reporter activity.

### *In vivo* transfection by electroporation

In vivo transfection was done by subretinal injection of reporter plasmids followed by electroporation as described [32]. Briefly, C57BL/6 mice (4–5 weeks old) were given a subretinal injection of 1µl of PBS containing 0.5 ug of pGL3 containing one of the firefly luciferase constructs described above and 0.25 ng of Renilla luciferase plasmid ( pRL-CMV, Promega, Madison, WI) for normalization. After injection, two steel electrodes separated by about 3.0 mm were placed on the posterior sclera and an ECM830 electroporator (BTX, San Diego, CA, USA) was used to deliver eight 50 ms electric pulses separated by 100 ms with voltage set at 30V. The eyes were enucleated 65–68h after electroporation and the cornea and lens were removed. Posterior eyecups were minced and homogenized in 100 µl of Reporter Lysis Buffer (Promega, Madison, WI). Firefly and Renilla luciferase activities were measured using 40µl of lysate and a Dual-Luciferase Reporter Assay System (Promega, Madison, WI). The firefly luciferase value was divided by the Renilla luciferase value to give the normalized luciferase activity. The normalized luciferase activity of each test plasmid injection group was divided by the normalized luciferase activity of empty-PGL3 and pRL-CMV injection group to give the relative luciferase activity ratio. Statistical analysis was done using ANOVA and Bonferroni/Dunn test.

## Supporting Information

Table S1 SNPs identified by re-sequencing.
(0.11 MB DOC)

Table S2 Primers for genotyping of SNPs.
(0.05 MB DOC)

## References

[1] Manolio TA, Brooks LD, Collins FS (2008). A HapMap harvest of insights into the genetics of common disease.. J Clin Invest.

[2] Zareparsi S, Branham KE, Li M, Shah S, Klein RJ (2005). Strong association of the Y402H variant in complement factor H at 1q32 with susceptibility to age-related macular degeneration.. Am J Hum Genet.

[3] Klein RJ, Zeiss C, Chew EY, Tsai JY, Sackler RS (2005). Complement factor H polymorphism in age-related macular degeneration.. Science.

[4] Haines JL, Hauser MA, Schmidt S, Scott WK, Olson LM (2005). Complement factor H variant increases the risk of age-related macular degeneration.. Science.

[5] Hageman GS, Anderson DH, Johnson LV, Hancox LS, Taiber AJ (2005). A common haplotype in the complement regulatory gene factor H (HF1/CFH) predisposes individuals to age-related macular degeneration.. Proc Natl Acad Sci U S A.

[6] Edwards AO, Ritter R, Abel KJ, Manning A, Panhuysen C (2005). Complement factor H polymorphism and age-related macular degeneration.. Science.

[7] Li M, Atmaca-Sonmez P, Othman M, Branham KE, Khanna R (2006). CFH haplotypes without the Y402H coding variant show strong association with susceptibility to age-related macular degeneration.. Nat Genet.

[8] Maller J, George S, Purcell S, Fagerness J, Altshuler D (2006). Common variation in three genes, including a noncoding variant in CFH, strongly influences risk of age-related macular degeneration.. Nat Genet.

[9] Gold B, Merriam JE, Zernant J, Hancox LS, Taiber AJ (2006). Variation in factor B (BF) and complement component 2 (C2) genes is associated with age-related macular degeneration.. Nat Genet.

[10] Yates JR, Sepp T, Matharu BK, Khan JC, Thurlby DA (2007). Complement C3 variant and the risk of age-related macular degeneration.. N Engl J Med.

[11] Maller JB, Fagerness JA, Reynolds RC, Neale BM, Daly MJ (2007). Variation in complement factor 3 is associated with risk of age-related macular degeneration.. Nat Genet.

[12] Fisher SA, Abecasis GR, Yashar BM, Zareparsi S, Swaroop A (2005). Meta-analysis of genome scans of age-related macular degeneration.. Hum Mol Genet.

[13] Jakobsdottir J, Conley YP, Weeks DE, Mah TS, Ferrell RE (2005). Susceptibility genes for age-related maculopathy on chromosome 10q26.. Am J Hum Genet.

[14] Rivera A, Fisher SA, Fritsche LG, Keilhauer CN, Lichtner P (2005). Hypothetical LOC387715 is a second major susceptibility gene for age-related macular degeneration, contributing independently of complement factor H to disease risk.. Hum Mol Genet.

[15] Schmidt S, Hauser MA, Scott WK, Postel EA, Agarwal A (2006). Cigarette smoking strongly modifies the association of LOC387715 and age-related macular degeneration.. Am J Hum Genet.

[16] Kanda A, Chen W, Othman M, Branham KE, Brooks M (2007). A variant of mitochondrial protein LOC387715/ARMS2, not HTRA1, is strongly associated with age-related macular degeneration.. Proc Natl Acad Sci U S A.

[17] Yang Z, Camp NJ, Sun H, Tong Z, Gibbs D (2006). A variant of the HTRA1 gene increases susceptibility to age-related macular degeneration.. Science.

[18] Dewan A, Liu M, Hartman S, Zhang SS, Liu DT (2006). HTRA1 promoter polymorphism in wet age-related macular degeneration.. Science.

[19] Fritsche LG, Loenhardt T, Janssen A, Fisher SA, Rivera A (2008). Age-related macular degeneration is associated with an unstable ARMS2 (LOC387715) mRNA.. Nat Genet.

[20] Gibbs D, Yang Z, Constantine R, Ma X, Camp NJ (2008). Further mapping of 10q26 supports strong association of HTRA1 polymorphisms with age-related macular degeneration.. Vision Res.

[21] Jakobsdottir J, Conley YP, Weeks DE, Ferrell RE, Gorin MB (2008). C2 and CFB genes in age-related maculopathy and joint action with CFH and LOC387715 genes.. PLoS ONE.

[22] Deangelis MM, Ji F, Adams S, Morrison MA, Harring AJ (2008). Alleles in the HtrA serine peptidase 1 gene alter the risk of neovascular age-related macular degeneration.. Ophthalmology.

[23] Hughes AE, Orr N, Patterson C, Esfandiary H, Hogg R (2007). Neovascular age-related macular degeneration risk based on CFH, LOC387715/HTRA1, and smoking.. PLoS Med.

[24] Seddon JM, Francis PJ, George S, Schultz DW, Rosner B (2007). Association of CFH Y402H and LOC387715 A69S with progression of age-related macular degeneration.. JAMA.

[25] Chan CC, Shen D, Zhou M, Ross RJ, Ding X (2007). Human HtrA1 in the archived eyes with age-related macular degeneration.. Trans Am Ophthalmol Soc.

[26] Francis PJ, Appukuttan B, Simmons E, Landauer N, Stoddard J (2008). Rhesus monkeys and humans share common susceptibility genes for age-related macular disease.. Hum Mol Genet.

[27] Singh KK, Krawczak M, Dawson WW, Schmidtke J (2009). Association of HTRA1 and ARMS2 gene variation with drusen formation in rhesus macaques.. Exp Eye Res.

[28] (1999). The Age-Related Eye Disease Study (AREDS): design implications. AREDS report no. 1.. Control Clin Trials.

[29] Abramson JH, Gahlinger PM (2001). PEPI, ver. 4.0: Computer Programs for Epidemiologists.

[30] Barrett JC, Fry B, Maller J, Daly MJ (2005). Haploview: analysis and visualization of LD and haplotype maps.. Bioinformatics.

[31] Gabriel SB, Schaffner SF, Nguyen H, Moore JM, Roy J (2002). The structure of haplotype blocks in the human genome.. Science.

[32] Kachi S, Oshima Y, Esumi N, Kachi M, Rogers B (2005). Nonviral ocular gene transfer.. Gene Ther.

