# Supplementary material for: Genetic and Functional Dissection of HTRA1 and LOC387715 in Age-Related Macular Degeneration
Source: PLoS Genet. 2010 Feb 5;6(2):e1000836. doi: 10.1371/journal.pgen.1000836 (PMC2816682; doi:10.1371/journal.pgen.1000836)
Supplement: Table S1 — SNPs identified by re-sequencing. (0.11 MB DOC) [file pgen.1000836.s001.doc]

**Table S1.** SNPs identified by re-sequencing

| **SNP No.** | **SNP ID** | **Gene** | **Role** | **Nucleotide Change** | **Amino Acid Change** | **Physical position** | **Frequency** | |
| --- | --- | --- | --- | --- | --- | --- | --- | --- |
|  |  |  |  |  |  |  | **Control** | **Case** |
| 1 | rs7350442 |  | Intergenic PLEKHA1- ARMS2 | T>C |  | 124182170 | 7/12 | 0/12 |
| 2 | rs10510110 |  | Intergenic PLEKHA1- ARMS2 | T>C |  | 124182420 | 1/12 | 12/12 |
| 3 | rs10510111 |  | Intergenic PLEKHA1- ARMS2 | A>C |  | 124182464 | 3/12 | 0/12 |
| 4 | rs2280141 |  | Intergenic PLEKHA1- ARMS2 | T>G |  | 124183171 | 4/12 | 12/12 |
| 5 | rs4752695 |  | Intergenic PLEKHA1- ARMS2 | G>C |  | 124185031 | 4/12 | 0/12 |
| 6 | SNP-1(New SNP) |  | Intergenic PLEKHA1- ARMS2 | T>C |  | 124187205 | 8/12 | 12/12 |
| 7 | SNP-2 (New SNP) |  | Intergenic PLEKHA1- ARMS2 | CATAAA>Del |  | 124198539 | 0/12 | 12/12 |
| 8 | rs11200630 |  | Intergenic PLEKHA1- ARMS2 | T>C |  | 124199674 | 0/12 | 12/12 |
| 9 | ENSSNP6019764 |  | Intergenic PLEKHA1- ARMS2 | G>A |  | 124200359 | 0/12 | 12/12 |
| 10 | rs10490924 | ARMS2 | Exon | G>T | S69A | 124204438 | 0/12 | 12/12 |
| 11 | SNP-3 (New SNP) | ARMS2 | Intron | Ins GT |  | 124204546-124204547 | 0/12 | 12/12 |
| 12 | rs36212731 | ARMS2 | Intron | G>T |  | 124204966 | 0/12 | 12/12 |
| 13 | rs36212732 | ARMS2 | Intron | A>G |  | 124205188 | 0/12 | 12/12 |
| 14 | rs36212733 | ARMS2 | Intron | T>C |  | 124205201 | 0/12 | 12/12 |
| 15 | rs3750848 | ARMS2 | Intron | T>G |  | 124205305 | 0/12 | 12/12 |
| 16 | rs3750847 | ARMS2 | Intron | C>T |  | 124205411 | 0/12 | 12/12 |
| 17 | rs3750846 | ARMS2 | Intron | T>C |  | 124205555 | 0/12 | 12/12 |
| 18 | SNP-4 (New SNP) | ARMS2 | Intron | Ins AT |  | 124206332-124206333 | 0/12 | 12/12 |
| 19 | in/del/Wt | ARMS2 | 3’-UTR | Ins 54bp, del 443bp |  | 124206811-124207253 | 0/12 | 12/12 |
| 20 | rs2014307 |  | Intergenic– ARMS2-HTRA1 | G>T |  | 124207622 | 9/12 | 0/12 |
| 21 | SNP-5 (New SNP) |  | Intergenic – ARMS2-HTRA1 | Ins C |  | 124207717-124207718 | 0/12 | 12/12 |
| 22 | rs5788557 |  | Intergenic – ARMS2-HTRA1 | Del>C |  | 124207747 | 8/12 | 12/12 |
| 23 | rs3037985 |  | Intergenic – ARMS2-HTRA1 | Del>AGAGAGA |  | 124207893 | 12/12 | 12/12 |
| 24 | rs34109041 |  | Intergenic – ARMS2-HTRA1 | Del>AGAG |  | 124207894 | 2/12 | 12/12 |
| 25 | rs3793917 | HTRA1 | Promoter | C>G |  | 124209265 | 0/12 | 12/12 |
| 26 | rs3763764 | HTRA1 | Promoter | A>G |  | 124210051 | 0/12 | 12/12 |
| 27 | rs11200638 | HTRA1 | Promoter | G>A |  | 124210534 | 0/12 | 12/12 |
| 28 | rs2672598 | HTRA1 | Promoter | C>T |  | 124210672 | 12/12 | 0/12 |
| 29 | rs1049331 | HTRA1 | Exon | C>T | A34A | 124211260 | 0/12 | 12/12 |
| 30 | rs2293870 | HTRA1 | Exon | G>T | G36G | 124211266 | 0/12 | 12/12 |
| 31 | rs932274 | HTRA1 | Intron | C>T |  | 124215354 | 2/12 | 3/12 |
| 32 | rs2284665 or rs57580727 | HTRA1 | Intron | G/T |  | 124216620 | 1/12 | 10/12 |
| 33 | rs61315710 | HTRA1 | Intron | Del>ATCATC |  | 124217944-124217949 | 6/12 | 0/12 |
| 34 | rs2672594 | HTRA1 | Intron | T>A |  | 124218192 | 1/12 | 0/12 |
| 35 | rs11200643 | HTRA1 | Intron | C>T |  | 124219193 | 1/12 | 12/12 |
| 36 | rs58077526 or rs61871752 | HTRA1 | Intron | A>C |  | 124220014 | 0/12 | 12/12 |
| 37 | rs2672593 or rs58005949 | HTRA1 | Intron | A>G |  | 124220096 | 0/12 | 0/12 |
| 38 | rs2672592 or rs57306740 | HTRA1 | Intron | G>T |  | 124220740 | 4/12 | 12/12 |
| 39 | rs2268344 | HTRA1 | Intron | T>C |  | 124234959 | 6/12 | 0/12 |
| 40 | rs2268345 | HTRA1 | Intron | G>T |  | 124235473 | 10/12 | 0/12 |
| 41 | rs878107 or rs58421672 | HTRA1 | Intron | C>T |  | 124235857 | 6/12 | 12/12 |
| 42 | rs34013554 | HTRA1 | Intron | Del>GT |  | 124236263-124236264 | 0/12 | 2/12 |
| 43 | rs2268348 | HTRA1 | Intron | C>T |  | 124236913 | 3/12 | 0/12 |
| 44 | rs2672582 or rs59035737 | HTRA1 | Intron | C>T |  | 124256490 | 2/12 | 8/12 |
| 45 | rs28414499 or rs58102299 | HTRA1 | Intron | G>A |  | 124256606 | 8/12 | 2/12 |
| 46 | rs2672583 or rs58489912 | HTRA1 | Intron | G>A |  | 124257077 | 2/12 | 10/12 |
| 47 | rs2672584 | HTRA1 | Intron | C>T |  | 124257233 | 9/12 | 2/12 |
| 48 | SNP-6 (New SNP) | HTRA1 | Intron | T>G |  | 124257240 | 9/12 | 12/12 |
| 49 | rs2736925 or rs57664551 | HTRA1 | Intron | C>G |  | 124258530 | 1/12 | 0/12 |
| 50 | rs28523390 | HTRA1 | Intron | G>A |  | 124260639 | 4/12 | 11/12 |
| 51 | rs2272599 | HTRA1 | Intron | A>G |  | 124261585 | 3/12 | 10/12 |
| 52 | rs12762192 | Downstream HTRA1 |  | A>G |  | 124268228 | 5/12 | 1/12 |
| 53 | rs2975745 | Downstream HTRA1 |  | G>A |  | 124271339 | 6/12 | 11/12 |
| 54 | rs12765507 | Downstream HTRA1 |  | C>T |  | 124274683 | 3/12 | 1/12 |
| 55 | rs11499280 | Downstream HTRA1 |  | C>A |  | 124275758 | 12/12 | 12/12 |
| 56 | rs11517384 | Downstream HTRA1 |  | T>A |  | 124278813 | 5/12 | 9/12 |
| 57 | rs7475480 | Downstream HTRA1 |  | A>T |  | 124280220 | 11/12 | 12/12 |
| 58 | rs3013210 | Downstream HTRA1 |  | C>T |  | 124280282 | 12/12 | 12/12 |
| 59 | rs7900422 | Downstream HTRA1 |  | A>C |  | 124280292 | 11/12 | 12/12 |
| 60 | rs3013211 | Downstream HTRA1 |  | T>C |  | 124280947 | 7/12 | 12/12 |
| 61 | rs11528742 | Downstream HTRA1 |  | C>A |  | 124281492 | 3/12 | 1/12 |
| 62 | rs4417210 | Downstream HTRA1 |  | C>T |  | 124282006 | 3/12 | 1/12 |
| 63 | rs11528744 | Downstream HTRA1 |  | T>C |  | 124283644 | 8/12 | 11/12 |
| 64 | rs11528736 | Downstream HTRA1 |  | C>T |  | 124284511 | 4/12 | 1/12 |
| 65 | rs11528745 | Downstream HTRA1 |  | A>G |  | 124284557 | 9/12 | 11/12 |
| 66 | SNP-7 (New SNP) | Downstream HTRA1 |  | A>G |  | 124284734 | 11/12 | 10/12 |
| 67 | rs4752704 | Downstream HTRA1 |  | G>A |  | 124284812 | 2/12 | 0/12 |
