# Supplementary material for: Genetic and Functional Dissection of HTRA1 and LOC387715 in Age-Related Macular Degeneration
Source: PLoS Genet. 2010 Feb 5;6(2):e1000836. doi: 10.1371/journal.pgen.1000836 (PMC2816682; doi:10.1371/journal.pgen.1000836)
Supplement: Table S2 — Primers for genotyping of SNPs. (0.05 MB DOC) [file pgen.1000836.s002.doc]

**Table S2.** Primers for genotyping of SNPs

| **Variants** | **Forward PCR primer** | **Reverse PCR primer** | **SnapShot primer** |
| --- | --- | --- | --- |
| SNP-2 (New SNP) | tacaagtgttgtaactatgt | ggactgataaacatgac | ggttcccctttttgtttttgataaa |
| ENSSNP6019764 | taccctgcgatctgatttcc | agatgcttgcagcagaaggt | aaaagtacctcgaccatgattcatcagg |
| rs1049331 | gagtcgccatgcagatcc | ctggcgcacacacagagg | ggccgctcggcgcctttggc |
| rs10664316 | tttggaaggagggggtctac | cttgctgcagtgtggatgat | atttaaagtgctcctcaacctaaaatatcgtcatgtgtct |
| rs11200630 | tttggtgtgaagcatgtggt | tgattccgaggaaattcagg | tgtccaggcagtagcttgaa |
| rs124198539 | ctaggccactctcgcttttg | atccaggagaaagggtccag | ccagtcgatggttcccctttttgtttttgataaa |
| rs2253755 | gtttccctgcacaagctctc | caaattgtttggcctccact | tctttatcagcagtgtgaaaatggactaatacagtagtgcagtcattttttc |
| in/del/Wt | cctgtcatcctgcctttgtt | gtagtttccaggggctctcc | ttcttgccctcctttctctcccgg |
| rs2293870 | ggccgctcggcgcctttggc | ccccgaagggcaccacgcac | cggccgctcggcgcctttggccgccgg |
| rs3793917 | ggtggtgaataaattggtg | agggaaagagcctagaaac | ccctgagcagccagactgcagagggaatga |
| rs3037985 | ctattcagaagcctcgttt | agtcttgctggaagggtt | aatggcagcagatggggaagaatctaaa |
| rs36212733 | taatgcttacggaactgt | tcaaggacattatgagcc | tttgtgcttgccatagtatatataa |
| rs36212732 | tattctcacggctttcca | acatgctgccatttaggc | taatgcttacggaactgtggcgctttgtgcttgcc |
| rs36212731 | aaacaagaagacgcagta | cagttccgtaagcattag | agtcatttagaaagctgtaccattctttcaatattctcac |
| rs22938702 | tcttctcccgctgctgct | acaggttggcgtaggtgttg | agcgcgccggctcgcagcggtctgggca |
| rs2672587 | gccccacaacactcatttct | atgtgcagtaccagcagcag | ccagcccagagcccggcccagcactggtctggggcatggaggcagcagaaacagaagc |
| rs2672598 | ggggaaagttcctgcaaatc | tcacttcactgtgggtctgg | ggcccctgcagtccctgcccggcccagtccgagc |
| rs714816 | aaccccaaagaaccagaacc | tgagggggtgacatctaagc | aaactagctaaccagaaacttgttctggagggttctaataaactca |
| rs932275 | gtccatgctcatggttttcc | ctcattccatgtgtccacca | aaacccttctccctacttctccctaaaataatttccttgg |
| rs3750846 | gctgctgctcagtttccttt | ttgaaccccttccctctctt | aacccttcccctaaatcagttgcatgag |
| rs3750847 | gctgctgctcagtttccttt | ttgaaccccttccctctctt | gacagaaattgacaagctgtcattcaagaccttt |
| rs3750848 | gctgctgctcagtttccttt | ttgaaccccttccctctctt | cagatttaaaattctggctcataatgtccttgattcaatg |
| rs2736911 | tacccaggaccgatggtaac | gtcactggctccttctccac | ggtccagcacagactctc |
| SNP-3 (New SNP) | gaaaactcatttctcactcacca | agggctgcagagaggatgt | ctgtttggcttcaaaactgccccc |
| SNP-4 (New SNP) | gctccagttggcttttaagg | tgctcctcaacctaaaatatcg | Gaaaaaaaaatagatttagtaatatgcatttttaaa |
| SNP-5 (New SNP) | aagcctcgtttcctgatt | ccttggcacggtattcta | ggaactcgtctgtttggcttcaaaactgccccc |
| rs58077526 | gctgcctaggagacatcagc | gccaactagaggtcctaatctctt | caattcctaatgcttcagtgctaggaatcctaaagcattaa |
